# Visualization of G3BP1–RNA Condensate Nascent Assembly and Early Maturation by HS-AFM

**DOI:** 10.3390/ijms27136052

**Published:** 2026-07-06

**Authors:** S. M. Neaz Mahmud, Noriyuki Kodera, Hanae Sato

**Affiliations:** 1 Division of Nano Life Science, Graduate School of Frontier Science Initiative, Kanazawa University, Kakuma-machi, Kanazawa 920-1192, Ishikawa, Japan; neazmahmud@stu.kanazawa-u.ac.jp; 2 WPI Nano Life Science Institute (NanoLSI), Kanazawa University, Kanazawa 920-1192, Ishikawa, Japan; nkodera@staff.kanazawa-u.ac.jp

**Keywords:** G3BP1, stress granule, HS-AFM

## Abstract

Stress granules (SGs) are stress-induced ribonucleoprotein condensates assembled around untranslated mRNAs and RNA-binding proteins. G3BP1 is a central regulator of SG formation, yet the molecular events that initiate G3BP1-mediated condensation remain poorly understood. Current models propose that condensation is initiated by RNA–RNA interactions, G3BP1 self-association, or RNA-dependent assembly of G3BP1 into higher-order networks. To define the earliest steps of condensate formation, we employed high-speed atomic force microscopy (HS-AFM) to monitor G3BP1–RNA assembly at the nanometer scale. HS-AFM revealed that G3BP1 first associates with RNA to form discrete nascent assemblies that progressively recruit additional RNA and G3BP1 molecules. These assemblies subsequently grow into higher-order RNA–protein condensates through stepwise assembly. Together, these observations identify RNA-bound G3BP1 assemblies as the initiating structures of condensate formation and provide a framework for understanding the early stages of stress granule assembly.

## 1. Introduction 

Biomolecular condensation is considered as a representative of cellular organization administrating diverse cellular functions [1,2]. These condensations are associated with RNA metabolism, protein complex assembly and cell-fate specification [3,4,5]. The condensates participated in RNA metabolism are considered as ribonucleoprotein (RNP) granules [6]. RNP granules are biomolecular condensates composed primarily of RNA and proteins and are found in both the nucleus (e.g., nucleoli, Cajal bodies, nuclear speckles, paraspeckles, promyelocytic leukemia bodies) and cytoplasm (e.g., P bodies, stress granules (SGs), and RNA transport granules) [7]. These condensates are associated with various diseases and dysfunction of condensates impacts neurodegeneration, infectious diseases and cancer [8,9,10].

SG is a distinguished type of RNP granule formed in the cytoplasm of eukaryotic cells in response to multiple stressors [11]. SG assembly is accompanied by translation-limiting conditions coupled with polysome disassembly, which results in an increase in uncoated mRNAs in the cytoplasm [12]. Several studies have confirmed the participation of thousands of different RNAs and hundreds of proteins in SG assembly, but the precise molecular mechanisms and exact contributions of individual components are still elusive [11,13]. SG assembly is thought to involve multivalent protein–protein, protein–RNA, and RNA–RNA interactions that collectively drive condensate formation [14,15,16]. In addition, several studies have confirmed the essential roles of several RNA-binding proteins, including TIA1 [17], G3BP1 and G3BP2 [18], CSDE1, PRRC2C [19], and UBAP2L [20], in different SG formations. During stress, G3BP dimers interact with a subset of non-translating mRNAs and other RNA-binding proteins, promoting the formation of multivalent interaction networks that initiate stress granule assembly. G3BP1 exhibits relatively broad RNA-binding specificity and can associate with numerous cellular transcripts.

G3BP1 is a central regulator of stress granule assembly and functions as a molecular switch that couples stress signaling to condensate formation. RNA binding relieves G3BP1 autoinhibition and promotes assembly of G3BP1-containing condensates [21,22,23,24]. However, the molecular events that initiate G3BP1-mediated condensation remain unclear. Current models propose that condensation may be driven primarily by intermolecular RNA–RNA interactions [13,25], by self-association of G3BP1 [21,26] or by RNA-dependent assembly of G3BP1 [16,27] into higher-order interaction networks. Distinguishing among these mechanisms requires direct observation of the earliest stages of condensate formation, which has remained challenging using conventional biochemical and imaging approaches.

High speed atomic force microscopy (HS-AFM) is a scanning probe microscopy (SPM) that enables direct visualization of biomolecular dynamics at nanometer spatial resolution and sub-second temporal resolution under near-physiological conditions. HS-AFM has been successfully applied to diverse biological systems, including molecular motor myosin V walking on actin filament [28], coil–coil structure of laminin protein [29], rotary catalysis of F1-ATPase [30], lipid membrane remodeling by ESCRT-III polymerization [31], and CRISPR Cas9-mediated DNA cleavage [32]. These capabilities make HS-AFM particularly well suited for examining the dynamic molecular events underlying condensate assembly.

To define the molecular events that initiate G3BP1-mediated condensation, we employed HS-AFM to visualize G3BP1–RNA assembly in real time at nanometer resolution. Our analysis revealed that G3BP1 forms a homodimer under near-physiological conditions and is associated with RNA to generate discrete initial assembly structures. These assemblies progressively recruit additional RNA and G3BP1 molecules, giving rise to higher-order condensates. Together, our findings provide a direct view of the earliest stages of stress granule assembly and establish a framework for understanding how G3BP1 organizes RNA during condensate formation.

## 2. Results

### 2.1. HS-AFM Reveals Dynamic Dimeric Architecture and pH-Dependent Compaction of G3BP1

**Figure 1:**
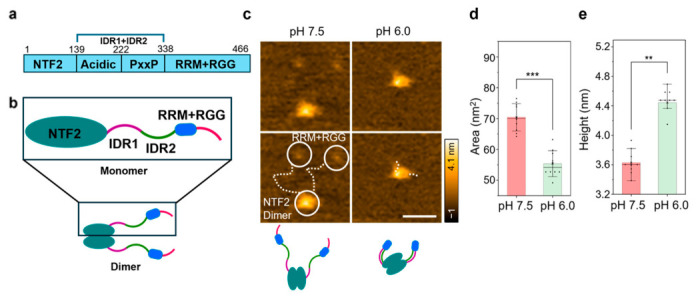
Structural features of G3BP1. (**a**) Domain architecture of G3BP1 protein. G3BP1 contains five motifs. (**b**) Schematic of G3BP1 structure. Approximate position of each domain is shown for both monomer and dimer. (**c**) HS-AFM frames showing real-time dimer structures of G3BP1 protein in both pH (7.5 and 6.0). Imaging was performed in an 80 × 80 nm scanning area with 80 × 80 pixels (scale bar, 20 nm). (**d**) Bar graph showing the mean condensate area of G3BP1 at pH 7.5 and pH 6.0. Data are presented as mean ± SD (*n* = 10). Statistical significance was determined using an independent *t*-test (*** *p* < 0.001). (**e**) Bar graph showing the average height of G3BP1 at pH 7.5 and pH 6.0. Data are presented as mean ± SD (*n* = 10). Statistical significance was determined using an independent *t*-test (** *p* < 0.01).

G3BP1 is a 466-amino-acid RNA-binding protein containing an N-terminal nuclear transport 2 (NTF2)-like domain that mediates homodimerization and a C-terminal RNA binding region comprising a canonical RNA recognition motif (RRM) and an arginine–glycine rich (RGG) box [33,34] (Figure 1a,b). The RRM of G3BP1 has RNP1 and RNP2 motifs separated by variable lengths and compositions of amino acids [35,36].

To examine the structural organization of G3BP1 under near-physiological conditions, we visualized purified G3BP1 using HS-AFM in scanning buffer at pH 7.5. Individual G3BP1 molecules appeared predominantly as dimers, consistent with previous biochemical studies [21,37]. HS-AFM imaging revealed a stable globular core associated with a smaller highly mobile globular domain (Figure 1c). The larger globular structure is consistent with the NTF2-mediated dimerization interface, whereas the mobile peripheral density likely corresponds to the C-terminal RNA-binding region connected through intrinsically disordered segments. Although the disordered regions were not clearly defined but remained faintly visible, the dynamic motion of the peripheral globular domain indicates substantial conformational flexibility of G3BP1 dimer under near-physiological conditions (Figure 1c).

Interestingly, the predominantly open and dynamic dimer conformation observed at pH 7.5 adopted a more compact conformation under acidic conditions (pH 6.0) (Figure 1c–e). Because the disordered regions are not visible, the precise dimer arrangement of G3BP1 at pH 6.0 cannot be definitively determined. Nevertheless, the increased particle height observed at pH 6.0 suggests that G3BP1 maintains a dimeric state while adopting a more compact conformation relative to that observed at pH 7.5 (Figure 1e). This pH-dependent conformational change is consistent with previous reports describing G3BP1 activation at lower pH and supports a model in which G3BP1 undergoes structural switching in response to changes in pH [37].

### 2.2. G3BP1 Promotes In Vitro RNA Condensation in a Concentration-Dependent Manner

**Figure 2:**
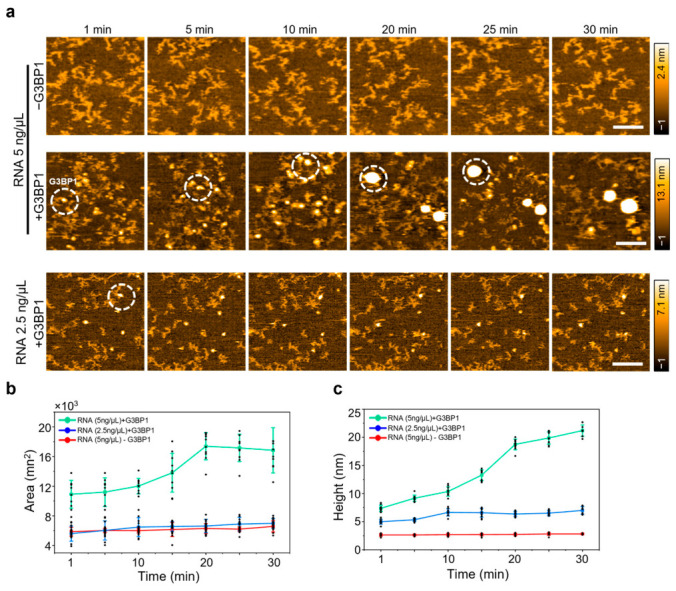
HS-AFM observation of droplet formation by total RNA and G3BP1. (**a**) HS-AFM images showing 5 ng/µL RNA with and w/o G3BP1 (10 nM) and 2.5 ng/µL RNA with G3BP1 (10 nM). Imaging was performed in a 350 × 350 nm scanning area with 100 × 100 pixels (Scale bar, 100 nm). (**b**) Time-course analysis of condensate area following G3BP1 addition to RNA. Measurements were performed under the experimental conditions shown in (**a**). Data are presented as mean ± SD. (**c**) Time-dependent changes in condensate height following G3BP1 addition to RNA. Measurements were acquired under the experimental conditions shown in (**a**). Data are presented as mean ± SD.

To examine how G3BP1 influences RNA condensation, we visualized mixtures of purified full-length G3BP1 and total RNA isolated from HEK293T cells using HS-AFM. In the absence of G3BP1, RNA molecules at 5 ng/µL appeared predominantly as isolated linear threads on the mica surface, with no evidence of higher-order assembly under the imaging conditions (Figure 2a, upper panel). Upon addition of G3BP1 (10 nM), protein molecules rapidly associated with RNAs, indicating robust RNA–protein interactions. Within 10 min, G3BP1–RNA complexes assembled into larger aggregates that appeared as microscale spherical structures or reticulated RNA–protein networks (Figure 2a, middle panel). These assemblies were highly dynamic and frequently interacted with nearby RNA molecules and protein complexes. Over time, continued accumulation of RNA and G3BP1 led to the formation of larger droplet-like condensates that became evident after approximately 30 min (Figure 2a, middle panel). These observations suggest that condensate formation proceeds through progressive growth of RNA-bound G3BP1 assemblies.

Importantly, G3BP1-driven condensation was highly dependent on RNA concentration. When the RNA concentration was reduced to 2.5 ng/µL while maintaining the same G3BP1 concentration (10 nM), G3BP1 remained capable of binding RNA; however, no higher-order aggregates or droplet-like condensates were observed even after 30 min of HS-AFM observation (Figure 2a, lower panel). Although HS-AFM imaging clearly visualized the RNA–protein interactions under this condition, these interactions are not enough for inducing RNA–RNA interactions that trigger phase separation. To further confirm this concentration dependence, we compared the area and height across three distinct sample conditions to assess morphological changes quantitatively. Both parameters increased following the addition of G3BP1 in both the 2.5 and 5 ng/µL RNA samples. However, no further changes in either metric were observed for the 2.5 ng/µL RNA samples, consistent with the absence of noticeable structural changes after G3BP1 addition (Figure 2b,c). Reflecting the droplet formation observed at 5 ng/µL, the area of these samples increased to approximately 17 × 10^3^ nm^2^ at 20 min post-protein addition before decreasing to 15 × 10^3^ nm^2^ at 30 min, likely due to condensate compaction during droplet formation (Figure 2b). The relatively large variation in area reflects the intrinsic heterogeneity of biomolecular condensate formation, which is commonly observed in phase separation. Similarly, the height of the 5 ng/µL aggregates gradually increased following G3BP1 addition, ultimately reaching approximately 21 nm at 30 min (Figure 2c). Conversely, the area and height of the 5 ng/µL RNA control without G3BP1 remained constant throughout the observation period (Figure 2b,c).

These findings demonstrate that in vitro biomolecular condensate formation is highly dependent on RNA concentration. Our data suggests that a critical density of RNA molecules is essential to trigger the higher-order assembly of G3BP1–RNA complexes under near-physiological conditions. This concentration-dependent accumulation likely facilitates the clustering of G3BP1, thereby driving phase separation.

### 2.3. Activation of G3BP1 Is Sufficient to Promote Condensation at Low RNA Concentrations

Previous studies have proposed that high concentrations of RNA activate G3BP1 by relieving its autoinhibited conformation, thereby triggering phase separation. However, it remains unclear whether high RNA concentrations are continuously required for subsequent network formation and condensation propagation after G3BP1 activation or whether their primary role is to induce the active state of the protein.

**Figure 3:**
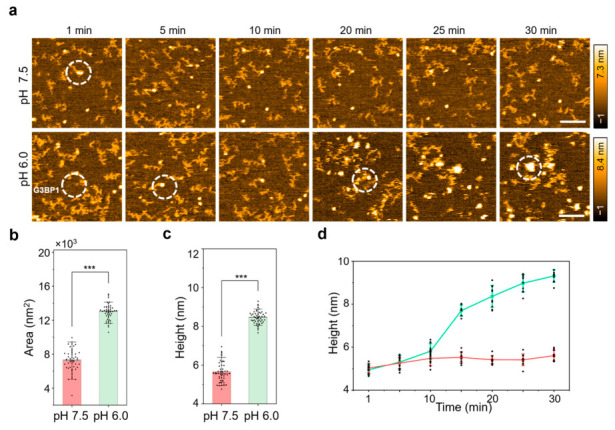
Activation of G3BP1 through pH. (**a**) HS-AFM image showing RNA and G3BP1 binding at pH 7.5 and 6.0. No aggregation was noticed after 30 min at pH 7.5 where noticeable aggregates were found at pH 6.0. Here, 2.5 ng/µL total RNA and 10 µM G3BP1 protein was used at room temperature. Imaging was performed in a 350 × 350 nm scanning area with 100 × 100 pixels (Scale bar, 100 nm). (**b**,**c**) Bar graph presenting differences in area and height for both samples at 30 min time point. Area and height were measured in experimental conditions (**a**). Bar graph presenting mean ± SD of samples. An independent sample *t*-test was performed to compare the means (*n* = 50; *** *p* < 0.001). (**d**) Time-dependent changes in condensate height at the indicated pH conditions following G3BP1 addition. Measurements were performed under the experimental conditions shown in (**a**). Data are presented as mean ± SD.

To distinguish between these possibilities, we took advantage of the observation that acidic pH promotes G3BP1 compaction, a conformation associated with its activated state. This approach was further motivated by previous studies showing that cellular stress can reduce intracellular pH and promote stress granule assembly, suggesting that pH-dependent regulation contributes to stress granule formation under physiological conditions [38]. We therefore performed HS-AFM imaging under a low RNA concentration (2.5 ng/µL), which is insufficient to induce condensation at neutral pH. While G3BP1 remained largely dispersed at pH 7.5 (Figure 3a, upper panel), lowering the pH to 6.0 induced the formation of distinct molecular clusters despite the limited RNA availability (Figure 3a, lower panel, Figure S1a). Quantitative analysis confirmed that both area and height increased significantly within 30 min at pH 6.0 following G3BP1 addition (Figure 3b–d).

Because acidic pH has been reported to promote not only G3BP1 compaction but also G3BP1 self-association and phase separation under certain conditions [16], we examined whether these pH 6.0 condensates could arise from protein self-assembly alone. However, HS-AFM imaging of purified G3BP1 (10 nM) without RNA showed that the protein remained predominantly dispersed under both pH 7.5 and 6.0 (Figure S1b,c). While previous studies reported pH-dependent G3BP1 self-condensation, those experiments employed substantially higher protein concentrations (~5 µM) compared to the 10 nM used here. These results indicate that pH-induced protein self-condensation does not occur under our conditions and cannot account for the observed assemblies. Instead, we observed dynamic interactions between these nascent clusters, which appeared to connect with one another via the RNA exposed on their surfaces. This observation suggests that cluster merging may be initiated by intermolecular RNA–RNA interactions between adjacent nascent assemblies, representing the initial step of condensate maturation (Movie S1).

Together, these findings demonstrate that a high RNA concentration is not essential for the initiation of G3BP1–RNA condensate once G3BP1 adopts an activated state. Instead, high RNA concentrations appear to function primarily by relieving G3BP1 autoinhibition to trigger assembly. Once activated, G3BP1 can bypass the requirement for high RNA abundance during the initial stages of condensation, whereas elevated RNA levels may subsequently facilitate the further growth and maturation of the network.

### 2.4. HS-AFM Visualization Reveals G3BP1-Centered Initiation and RNA-Driven Condensate Maturation

To investigate how G3BP1–RNA condensates mature after nascent assembly formation, we monitored condensate dynamics using HS-AFM. Condensates were initially assembled using 5 ng/µL RNA and 10 nM G3BP1 in scanning buffer. After condensate formation, 100 mM NaCl was added to increase the ionic strength of the solution and examine its effect on the structural evolution of the preformed condensates.

**Figure 4:**
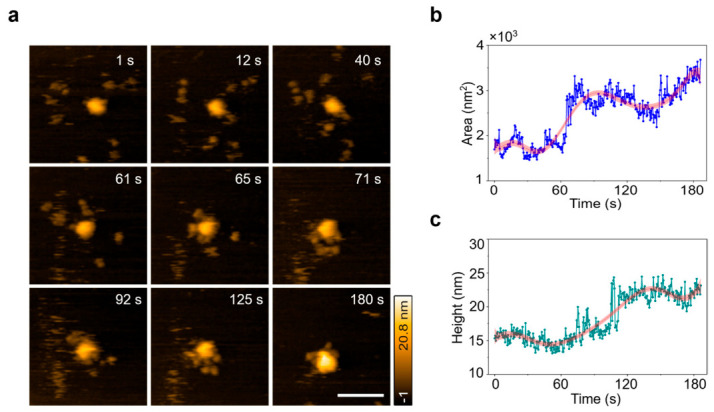
HS-AFM observation of droplet aging. (**a**) HS-AFM image showing the real-time condition of droplet maturation. Here, 5 ng/µL total RNA and 10 nM G3BP1 protein were used at room temperature. Imaging was performed in a 250 × 250 nm scanning area with 100 × 100 pixels (scale bar, 100 nm). (**b**) Line graph showing area of droplets over time under the condition in (**a**). The solid line represents a nonlinear regression fit to the experimental data, illustrating the trend in droplet area during maturation. (**c**) Line graph showing changes in height under the condition in (**a**). Height increases over time, and the solid line represents a nonlinear regression fit to the experiment data, illustrating the trend of height during maturation.

HS-AFM imaging revealed progressive enlargement of pre-existing condensates following NaCl addition (Figure 4a and Movie S2). Rather than the appearance and merger of multiple G3BP1-rich assemblies, condensate growth occurred around a dense pre-existing structure that remained visible throughout the observation period, suggesting that increased ionic strength promotes the maturation of preformed condensates. Quantitative analysis demonstrated a nonlinear increase in both condensate area and height over time (Figure 4b,c), indicating continuous condensate maturation. The increase in droplet dimensions was accompanied by an increase in molecular density within the condensate, suggesting progressive structural reorganization.

These observations provide insight into the mechanism of G3BP1-mediated condensation. Previous models proposed that RNA activates G3BP1, that a critical RNA–protein interaction network is required for phase separation, or that RNA promotes intermolecular G3BP1 assembly. In contrast, our HS-AFM data supports a model in which activated G3BP1 first establishes a stable nascent assembly center. Subsequent condensate growth is then achieved through the accumulation and organization of RNA around this core rather than through continued formation of new G3BP1 assemblies. Throughout the maturation process, the central G3BP1-rich structure remained preserved while the surrounding condensate progressively expanded. The nonlinear increase in condensate dimensions is consistent with concentration-dependent viscoelastic aging of biomolecular condensates [39,40]. Together, these HS-AFM observations suggest that G3BP1 acts as a nucleating seed for condensate formation, while condensate maturation is driven primarily by RNA accumulation around the established core.

## 3. Discussion

The molecular events that initiate G3BP1-mediated stress granule assembly have remained difficult to resolve because the earliest stages of condensate formation occur below the spatial resolution of conventional light microscopy and are often obscured in bulk biochemical measurements. By combining purified G3BP1 with HS-AFM imaging, we directly visualized dynamic interactions between G3BP1 and RNA at single-molecule resolution and followed the progression of condensate assembly in real time. Importantly, this approach enabled direct observation of the initiation events and emergence of condensate cores before they became detectable by conventional microscopy, providing molecular-level insight into the initiation of G3BP1-driven condensate assembly.

Consistent with previous SAXS and DLS studies [37], our HS-AFM analyses demonstrated that G3BP1 exists predominantly as a homodimer under near-physiological conditions, providing a direct visualization of its dynamic architecture in solution. Although HS-AFM enabled visualization of the overall architecture and assembly behavior of G3BP1, capturing the molecular details of RNA recognition remains challenging. Because intrinsically disordered regions (IDRs) generally elude conventional structural determination due to their high flexibility, the extensive IDRs of G3BP1 precluded the direct visualization of how the protein initially engages RNA and what conformational changes accompany its activation. Nevertheless, HS-AFM was able to capture the earliest detectable G3BP1–RNA assemblies and follow their progression into condensate cores, providing direct insight into the initiation process underlying condensate formation.

**Figure 5:**
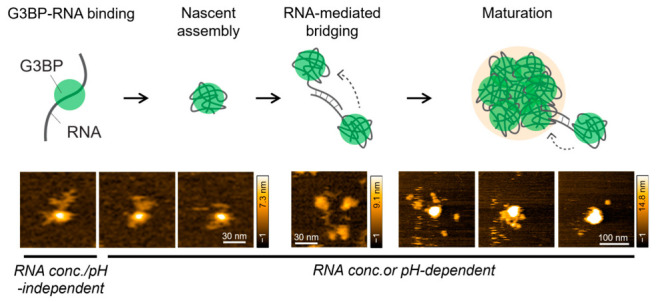
Model describes G3BP1–RNA condensation formation.

Our observations also provide insight into current models of G3BP1-mediated condensate formation. Previous studies have proposed that stress granule assembly may be initiated by RNA–RNA interactions that subsequently recruit G3BP1 [37,38,39], by self-association of G3BP1 followed by RNA recruitment [15,16,26,40], or by RNA-dependent assembly of G3BP1 into higher-order networks [16,22,26,41]. HS-AFM imaging directly visualized the formation of RNA-bound G3BP1 nascent assembly centers at the earliest detectable stage of assembly. We did not observe the formation of G3BP1 clusters in the absence of RNA or under non-activating conditions, indicating that RNA association and G3BP1 activation are closely coupled to the initiation of condensate assembly. Following nascent assembly, these assemblies progressively recruited additional RNA molecules and neighboring complexes to generate larger condensates. These observations identify RNA-bound G3BP1 assemblies as nucleation intermediates during condensate formation. Based on these findings, we propose a model in which activated G3BP1 forms nascent assembly that subsequently recruits additional RNA and protein molecules to drive condensate growth (Figure 5).

The concentration dependence of condensate formation further supports this model. Although RNA–G3BP1 interactions were readily detected at low concentrations, stable condensates formed only after sufficient accumulation of RNA and G3BP1 within growing assemblies, indicating a concentration-dependent condensate formation. Notably, condensates expanded through continuous recruitment of surrounding molecules but exhibited limited fusion with neighboring clusters, suggesting that condensate growth is constrained by the architecture of the underlying RNA–protein network.

Our pH-dependent experiments provide additional insight into the regulation of condensate initiation. Lowering the pH induced structural compaction of G3BP1, consistent with previous studies [38] and promoted the formation of RNA–protein clusters even at relatively low RNA concentrations. In contrast, under near-physiological pH conditions, substantially higher RNA concentrations were required to initiate cluster formation. Together, these findings indicate that low-pH-induced activation of G3BP1 facilitates early-stage condensate formation, thereby driving the initiation of the assembly process.

Although our study defines the early stage of condensate assembly and reveals key features of condensate growth, several limitations should be considered. G3BP1 was examined at a concentration of 10 nM, which is lower than the estimated physiological concentrations of G3BP1 (~250 nM to ~624 μM depending on cell type and measurement approach) [42,43]. This lower concentration was chosen to enable direct visualization of individual G3BP1 molecules and their dynamic behavior by HS-AFM while still supporting condensate formation under the experimental conditions used in this study. Nevertheless, our observations provided clues regarding the interactions that may contribute to condensate maturation. Approaching nascent assembly centers frequently appeared to establish contacts through peripheral RNA-containing structures, suggesting that intermolecular RNA–RNA interactions may contribute to the initial stages of condensate merging. Consistent with this interpretation, increasing the ionic strength by adding 100 mM NaCl after condensate formation promoted the maturation of preformed G3BP1–RNA assemblies, as revealed by enhanced interconnection between neighboring assemblies and the formation of larger condensates in HS-AFM images (Movie S2). This observation suggests that under the conditions tested, increased ionic strength facilitates the reorganization of preformed assemblies, possibly by reducing electrostatic repulsion between RNA-containing assemblies and promoting RNA-mediated interactions [44]. However, the relative contributions of RNA–RNA and G3BP1-G3BP1 interactions during subsequent condensate growth and maturation remain to be determined.

In summary, our study provides direct real-time visualization of the earliest stages of G3BP1-mediated condensate assembly at single-molecule resolution. By capturing the formation of RNA-bound G3BP1 nascent assembly, we establish a framework for understanding how activated G3BP1 initiates stress granule assembly and drives the emergence of higher-order RNA–protein condensates. More broadly, our work highlights the power of HS-AFM to resolve molecular events underlying biomolecular condensate initiation that are inaccessible to conventional imaging approaches.

## 4. Materials and Methods

### 4.1. Total RNA Extraction

Total RNA was extracted from human HEK 293T cells. Before RNA extraction 2.2 × 10^6^ cells were grown with Dulbecco’s modified Eagle’s medium (DMEM) containing 10% fetal bovine serum (FBS), 10,000 U/mL penicillin, and 10,000 µg/mL streptomycin sulfate (Gibco, Grand Island, NY, USA) in a cell incubator at 37 °C with 5% CO_2_. When fully confluent the total RNA was extracted using NucleoSpin RNA plus kit (Macherey-Nagel, Düren, Germany) following manufactural protocol.

### 4.2. Protein Preparation

Stock solution (0.5 mg/mL) of full-length human recombinant G3BP1 was purchased from Abcam (ab103304, Cambridge, UK). Stock solution was aliquoted and stored at −80 °C and diluted with the imaging buffer immediately in required concentration before adding to scanning chamber for HS-AFM imaging.

### 4.3. Cantilever Preparation

We used small cantilevers (BLAC10DS-A2, Olympus, Fukushima, Japan) with a nominal spring constant of ~0.1 N/m, resonant frequency of ~0.5 MHz, and a quality factor of ~1.5 in liquid. An amorphous carbon tip was fabricated on the original AFM tip by electron beam deposition. The length of the additional AFM tip was ~500 nm, and the tip was further sharpened using a radio-frequency plasma etcher (Tergeo, PIE Scientific LLC, Union City, CA, USA) under an argon gas atmosphere (Direct mode, 10 sccm, and 20 W for 1.5 min).

### 4.4. HS-AFM Imaging

A laboratory-built high-speed atomic force microscope [45] was used as described previously [46]. The samples were deposited on a glass sample stage (diameter, 2 mm; height, 2 mm) with a thin mica disk (1.5 mm in diameter and ~0.05 mm in thickness) glued to the top by epoxy and attached onto the top of a Z-scanner by a drop of nail polish.

After rinsing the surface five times with 20 µL of MilliQ-water, the surface was further rinsed with 20 µL of the imaging buffer [10 mM Tris-HCl (pH 7.5 or 6.0), 50 mM NaCl, 60 mM KCl], during which the surface was not allowed to dry. Then, 2 µL of RNA sample diluted with the imaging buffer was applied to the surface for 3 min. After rinsing the surface with 20 µL of the imaging buffer, the surface was immersed in the imaging chamber containing 65 µL of the scanning buffer and then imaged with HS-AFM in the tapping mode. The free oscillation amplitude of the cantilever was 2–3 nm, and the set-point amplitude was set to 90% of the free amplitude. G3BP1 was added to the imaging chamber to obtain the desired final concentrations. All HS-AFM experiments were performed at 24–26 °C, and data were collected using laboratory developed software UMEX (version 2026).

### 4.5. HS-AFM Image Processing

The HS-AFM images are processed in UMEX Viewer software where image contrast was normalized as needed for clear visualization. The mean plane subtraction, row alignment, and the correction of horizontal scan lines were applied if needed. The scale bar and height scale bar were generated in UMEX viewer software automatically. Movies were also generated in UMEX viewer software at 30 frames per second speed.

### 4.6. Data Analysis

The area and height of objects in HS-AFM images were measured and analyzed using UMEX Viewer. For height analysis, highest points were considered for all measurements. All statistical analysis was performed in Origin Pro 2025b software. A significant cutoff value *p* < 0.05 was used for all analyses unless otherwise noted. The normality of distribution was tested by the Shapiro–Wilk test. Tukey’s test was used for mean comparison.

## Data Availability

The original contributions presented in the study are included in the article/Supplementary Materials. Further inquiries can be directed to the corresponding authors.

## Supplementary Materials

The following supporting information can be downloaded at: https://www.mdpi.com/article/10.3390/ijms27136052/s1.
