# Supplementary material for: Visualization of G3BP1–RNA Condensate Nascent Assembly and Early Maturation by HS-AFM
Source: Int J Mol Sci. 2026 Jul 6;27(13):6052. doi: 10.3390/ijms27136052 (PMC13361221; doi:10.3390/ijms27136052)
Supplement: Supplementary file 1 [file ijms-27-06052-s001.zip › Movie Caption.pdf]

Movie S1. HS-AFM movie shows dynamic interactions between nucleation clusters. Scanning area is 500 nm × 500 nm with 120 × 120 pixels. The imaging rate was ~0.8 frames per second (fps), and the movie is played at ~30 fps. The contrast of the movie is -1 nm to 8.1 nm: scale bar, 100 nm.

Movie S2. HS-AFM movie showing G3BP1-RNA aging. Representative frames are presented in Figure 4a. Scanning area is 250 nm × 250 nm with 100 × 100 pixels. The imaging rate was ~1.9 frames per second (fps), and the movie is played at ~30 fps. The contrast of the movie is -1 nm to 20.8 nm: scale bar, 100 nm.
