# Supplementary material for: Visualization of G3BP1–RNA Condensate Nascent Assembly and Early Maturation by HS-AFM
Source: Int J Mol Sci. 2026 Jul 6;27(13):6052. doi: 10.3390/ijms27136052 (PMC13361221; doi:10.3390/ijms27136052)
Supplement: Supplementary file 1 [file ijms-27-06052-s001.zip › Supplementary figure.pdf]

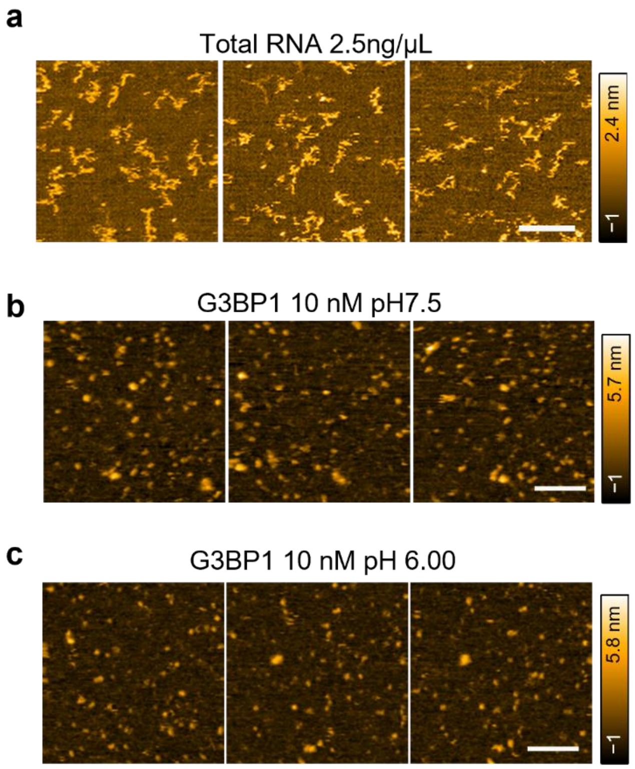

Figure S1

(a). HS-AFM images showing total RNA on mica surface without G3BP1. Here, 2.5 ng/μL toral RNA was used at room temperature. Imaging was performed in a 350 × 350 nm scanning area with 100× 100 pixels (Scale bar, 100 nm).

(b). HS-AFM images of G3BP1 acquired at pH 7.5. G3BP1 was imaged at a concentration of 10 nM at room temperature. HS-AFM imaging was performed over a 350 × 350 nm scan area with a resolution of 100 × 100 pixels. Scale bar, 100 nm.

(c). HS-AFM images of G3BP1 at pH 6.0. G3BP1 was imaged at a concentration of 10 nM at room temperature. Imaging was performed in a 350 × 350 nm scanning area with 100× 100 pixels (Scale bar, 100 nm).
